# Reduced photoreceptor layer thickness in early type 2 diabetes mellitus is associated with decreased contrast sensitivity: a prospective cross-sectional study

**DOI:** 10.3389/fmed.2026.1984894

**Published:** 2026-10-27

**Authors:** Yuanyuan Tu, Xuancheng Zhu, Yake Sun, Xiaohui Sun, Gang Cao

**Affiliations:** 1 Department of Ophthalmology, Lixiang Eye Hospital of Soochow University, Suzhou, Jiangsu, China; 2 Department of Ophthalmology, The Second Affiliated Hospital of Soochow University, Suzhou, Jiangsu, China; 3 Department of Ophthalmology, Laizhou People’s Hospital, Yantai, Shandong, China

**Keywords:** contrast sensitivity, diabetic retinopathy, photoreceptor, retina, T2DM

## Abstract

**Purpose:**

To investigate changes in contrast sensitivity (CS) in patients with Type 2 Diabetes Mellitus (T2DM) with no diabetic retinopathy (DR) or nonproliferative diabetic retinopathy (NPDR) and to assess the relationships between these alterations and early retinal microstructure.

**Methods:**

This prospective cross-sectional study included subjects aged 40–60 years, including 30 healthy controls, 30 individuals with T2DM without DR, and 30 individuals with T2DM and NPDR. General data from all groups were recorded. Contrast sensitivity was assessed using the CSV-1000E chart, and AULCSF was calculated as a summary measure of overall contrast sensitivity. Optical coherence tomography (OCT) and OCT angiography (OCTA) were used to analyze the thickness as well as blood flow of retinal sublayers. Differences in inflammatory factors, including PLR (PLT-to-lymphocyte ratio), SII (Systemic Immune-Inflammation Index), and NLR (Neutrophil-to-lymphocyte ratio), were compared among the three groups. Pearson correlation analyses were performed to assess the associations between contrast sensitivity, retinal structural parameters, and inflammatory markers. Exploratory ROC curve analysis was performed to evaluate the ability of photoreceptor layer thickness to identify control-defined contrast sensitivity abnormalities in participants with T2DM.

**Results:**

Significant between-group differences were observed in AULCSF, middle-spatial-frequency CS, photoreceptor layer thickness, and PLR. GCIPL thickness was reduced in patients with NPDR. No significant differences were observed in total, inner, or outer retinal thickness, FAZ, VD, AFI, SII, or NLR. Middle CS was positively correlated with photoreceptor thickness and PLR. Additionally, photoreceptor thickness differed significantly between DM patients with normal and abnormal CS, and Pearson correlation analysis confirmed a positive association between photoreceptor thickness and middle CS. The AUC for identifying abnormal CS among participants with T2DM based on photoreceptor layer thickness was 0.920 (95% CI: 0.852–0.989), indicating good discriminatory performance within the present study cohort.

**Conclusion:**

This study identified an association between retinal microstructure and visual function in individuals with T2DM. These findings suggest that CS abnormalities and reduced photoreceptor layer thickness are associated with early structural and functional retinal alterations before clinically detectable DR. Longitudinal studies are required to determine whether these parameters predict future DR development.

## 1 Introduction

Diabetic retinopathy (DR) is a common complication of type 2 diabetes mellitus (T2DM), which may cause vision impairment and blindness when left untreated (1). Retinopathy poses a significant public health challenge, affecting 34.6% of an estimated 415 million people with T2DM globally (2). DR is clinically categorized into two distinct forms: non-proliferative diabetic retinopathy (NPDR) and proliferative diabetic retinopathy (PDR), which are determined by the presence or absence of retinal neovascularization (3). NPDR progression can lead to PDR, which may cause visual impairment. Although the application of anti-VEGF agents and photocoagulation has reduced neovascularization and diabetic macular edema (DME), there are still many limitations (4, 5). Hence, researchers are now focusing on the early identification of DR. Early detection of DR could enable the implementation of effective strategies to prevent its progression.

In the absence of vascular changes or signs of inflammation, evaluating neuronal retinal integrity through clinical fundus examination is challenging. Still, it can be efficiently assessed using functional testing and imaging techniques. Contrast sensitivity (CS) is the inverse of the threshold contrast level at which the human visual system can detect a stimulus across various spatial frequencies. This measure offers a more thorough assessment of visual function compared to the conventional visual acuity charts (6, 7). Previous studies have reported abnormal CS in patients with T2DM with or without DR (8). Compared with patients with mild NPDR, those with moderate NPDR had lower AULCSF values, indicating reduced overall contrast sensitivity. Furthermore, participants without DR also showed lower AULCSF values than the control group (9). In this work, CS was conducted to measure visual function in patients with T2DM and DR.

Several studies have indicated that the early pathogenesis of DR involves retinal vasculature and neuronal tissues. Strong evidence suggests that the neural aspects of the retina and the photoreceptors are the initial sites of pathological changes, triggering the chain of events that lead to the characteristic microvascular manifestations of DR (10, 11). Technologies like optical coherence tomography (OCT) enable clinicians to visualize and assess retinal architecture *in vivo* non-invasively. These technologies can accurately and efficiently identify photoreceptors (12).

However, numerous studies focus exclusively on the changes in retinal microstructure and visual function in individuals with DR, with a lack of literature examining the relationship between these microstructural alterations and visual performance. Hence, this research aims to determine whether the changes in the microstructure of the photoreceptors in patients with T2DM-related retinopathy are associated with CS.

## 2 Methods

### 2.1 Subjects

This prospective cross-sectional study was conducted at the Department of Ophthalmology, Lixiang Eye Hospital of Soochow University. Participants aged 40 to 60 years were consecutively recruited from the ophthalmology outpatient clinic between January 2023 and December 2025. Eligible participants were enrolled according to predefined inclusion and exclusion criteria and were subsequently divided into three groups based on clinical evaluation and fundus photography: non-diabetic individuals (control group), individuals with T2DM without DR (NDR group), and individuals with T2DM and NPDR (NPDR group). The right eye was chosen for analysis in the control and NDR groups. Conversely, in the NPDR group, the eye exhibiting the disease was selected; if both eyes were affected, the right eye was chosen for study. All participants in the NPDR group had mild NPDR, and no moderate or severe NPDR cases were included in this study. The research flow chart is shown in Figure 1. The inclusion criteria for the control group were (1) the absence of a clinical diagnosis of diabetes and (2) the absence of detectable retinopathy. The inclusion criteria for the NDR group were (1) a clinical diagnosis of T2DM (13) and (2) absence of detectable retinopathy. The inclusion criteria for the NPDR group were (1) a clinical diagnosis of type 2 diabetes mellitus and (2) confirmation of mild NPDR through fundus examination. The diagnosis and severity classification of DR were based on the International Clinical Diabetic Retinopathy Disease Severity Scale. Participants with diabetic macular edema detected by fundus examination or OCT were excluded. All participants had good exam compliance and were not gender restricted. All participants were Chinese.

**Figure 1:**
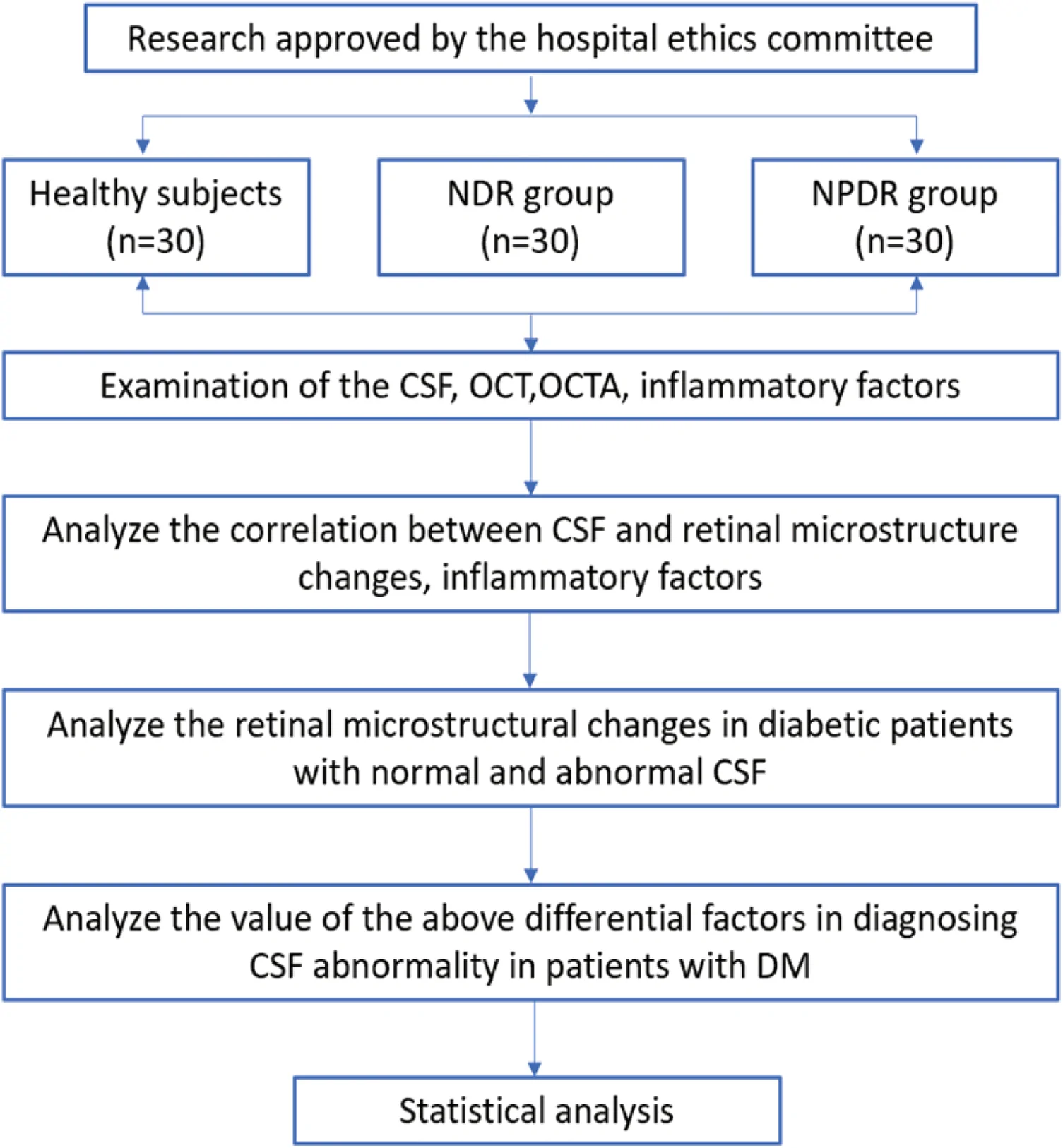
Flow chart of the study.

The exclusion criteria for all groups encompassed (1) the existence of severe systemic diseases aside from T2DM, hypertension, and hyperlipidemia (e.g., severe renal failure, malignancies); (2) PDR; (3) any ocular condition that could compromise vision, including cataracts, glaucoma, diabetic macular edema (DME), and other ocular fundus disorders, such as epiretinal membrane of the macula, retinal detachment, and vitreous hemorrhage; (4) coronary heart disease, stroke, aortic dissection, pulmonary embolism, and other severe cardiovascular and cerebrovascular conditions; (5) pregnancy or lactation; (6) inability to provide informed consent or to complete assessments. This study was approved by the Lixiang Eye Hospital Ethics Committee. The experiments were performed with the informed consent in writing of each participant.

The primary outcome was the association between middle-spatial-frequency contrast sensitivity and photoreceptor layer thickness. Secondary outcomes included alterations in OCTA microvascular metrics and systemic inflammatory indices.

### 2.2 Data collection

#### 2.2.1 Clinical data

General information was collected from the three groups, including age, gender, smoking status, alcohol consumption, presence of T2DM, T2DM duration since initial diagnosis, and presence of hypertension and hyperlipidemia. Laboratory parameters included glycated hemoglobin (HbA1c), aspartate aminotransferase (AST), alanine aminotransferase (ALT), platelet count, neutrophil count, and lymphocyte count. Best-corrected visual acuity (BCVA) was assessed after objective and subjective refraction using an international standard visual acuity chart. Visual acuity was recorded in decimal notation and converted to logarithm of the minimum angle of resolution (logMAR) units for statistical analysis. Intraocular pressure (IOP) was measured non-invasively in patients using a non-contact tonometer, while axial length was determined utilizing an optical biometer. The condition of the lens was examined using a slit lamp and cataract severity was graded using the Lens Opacities Classification System III (LOCS III).

#### 2.2.2 Inflammatory factors

Hematological and biochemical assessments were performed following an 8-h fast, with blood samples obtained through a forearm venipuncture. Following the standard protocols, the complete blood count was analyzed using a Sysmex XE-2100 system (Sysmex Corp., Kobe, Japan).

The systemic immune-inflammatory index (SII) was calculated by multiplying the platelet count by the neutrophil count and dividing the product by the lymphocyte count. The neutrophil-to-lymphocyte ratio (NLR) was obtained by dividing the neutrophil count by the lymphocyte count. Likewise, the platelet-to-lymphocyte ratio (PLR) was determined by dividing the platelet count by the lymphocyte count.

#### 2.2.3 Contrast sensitivity (CS)

Contrast sensitivity was assessed using the CSV-1000E chart (VectorVision, USA) after best-corrected visual acuity (BCVA) testing. Refractive correction was determined by objective refraction followed by subjective refinement. Testing was performed monocularly at a distance of 2.5 m, with the fellow eye occluded. Standardized photopic conditions were maintained throughout the examination. Background luminance followed the manufacturer's recommendations, and ambient illumination was kept constant. Participants were given sufficient time to adapt before testing.

The CSV-1000E chart assesses contrast sensitivity at four fixed spatial frequencies: 3, 6, 12, and 18 cycles/degree (cyc/deg). At each frequency, participants identified the location of sine-wave gratings presented at progressively lower contrast levels. The lowest correctly identified contrast level was recorded as the threshold. Thresholds were converted to logarithmic contrast sensitivity (logCS) using the manufacturer's scoring scale. No adaptive qCSF algorithm was used.

The area under the log contrast sensitivity function (AULCSF) was calculated from the measured logCS values across the tested logarithmic spatial-frequency range. It was used as a summary measure of overall contrast sensitivity. Low-, middle-, and high-spatial-frequency CS corresponded to 3, 6–12, and 18 cyc/deg, respectively. All examinations were performed by the same trained examiner, who was blinded to participants' diabetic status and clinical grouping.

#### 2.2.4 OCT and optical coherence tomography angiography examination

An ophthalmic technician performed OCT and optical coherence tomography angiography (OCTA) examinations in all three groups. OCTA was used to assess the foveal avascular zone (FAZ), vascular density (VD), and amplitude-decorrelation Fourier-domain imaging (AFI) of the superficial capillary plexus (SCP) and deep capillary plexus (DCP). Each examination was repeated three times, and the average values were recorded.

All OCT and OCTA images were acquired using a TowardPi swept-source OCTA system (TowardPi Medical Technology, Beijing, China). The system uses a 1,060 nm swept-source laser with an acquisition speed of 400,000 A-scans/s, providing an axial optical resolution of ≤6 μm and a transverse optical resolution of 10 μm. A 6 × 6 mm scanning protocol centered on the fovea was applied for all participants.

OCT/OCTA examinations were performed under standardized imaging conditions. Participants were instructed to maintain stable fixation using the internal fixation target of the device during image acquisition. Routine pupil dilation was not performed before OCT/OCTA examination. Eye-tracking and follow-up registration functions available in the imaging system were not specifically applied in this cross-sectional acquisition protocol.

Image quality was assessed before quantitative analysis. Images were considered acceptable when they demonstrated sufficient signal strength, clear visualization of retinal layer boundaries, and absence of significant motion artifacts. Images with a signal strength index <50, significant motion artifacts, or incorrect segmentation were excluded. Automated segmentation boundaries were reviewed by an experienced ophthalmologist before analysis, and manual correction was performed when segmentation errors were identified.

Key structural and vascular parameters were defined and measured as follows: Photoreceptor layer thickness (PRT) was defined as the vertical distance between the external limiting membrane (ELM) and the inner boundary of the retinal pigment epithelium (RPE). This OCT-derived parameter represents the photoreceptor-related outer retinal compartment, including the photoreceptor inner and outer segment regions, while excluding the outer nuclear layer (ONL) and RPE–Bruch's membrane complex. Because this measurement reflects a composite structural thickness rather than direct photoreceptor cell quantification, it was interpreted as photoreceptor layer thinning rather than photoreceptor cell loss. The inner and outer segment components were not separately quantified in this study.

For all participants, PRT was manually measured on the horizontal B-scan passing through the foveal center using the built-in caliper tool. The foveal center was automatically identified by the OCT system and manually confirmed before measurement. All PRT measurements were performed by the same examiner. No formal intraobserver or interobserver repeatability assessment was conducted, and the examiner's masking status was not documented. Ganglion cell-inner plexiform layer (GCIPL) thickness was automatically calculated using the built-in ganglion cell analysis protocol, representing the combined thickness of the ganglion cell layer and inner plexiform layer (GCL+IPL) within the macular annulus, without including the inner nuclear layer (INL). Cell density (CD) and cell regularity (CR) were also recorded as exploratory indices. These parameters were not interpreted as direct measurements of photoreceptor cell number or spatial organization. The measurement locations of GCIPL and PRT are illustrated in Figure 2.

**Figure 2:**
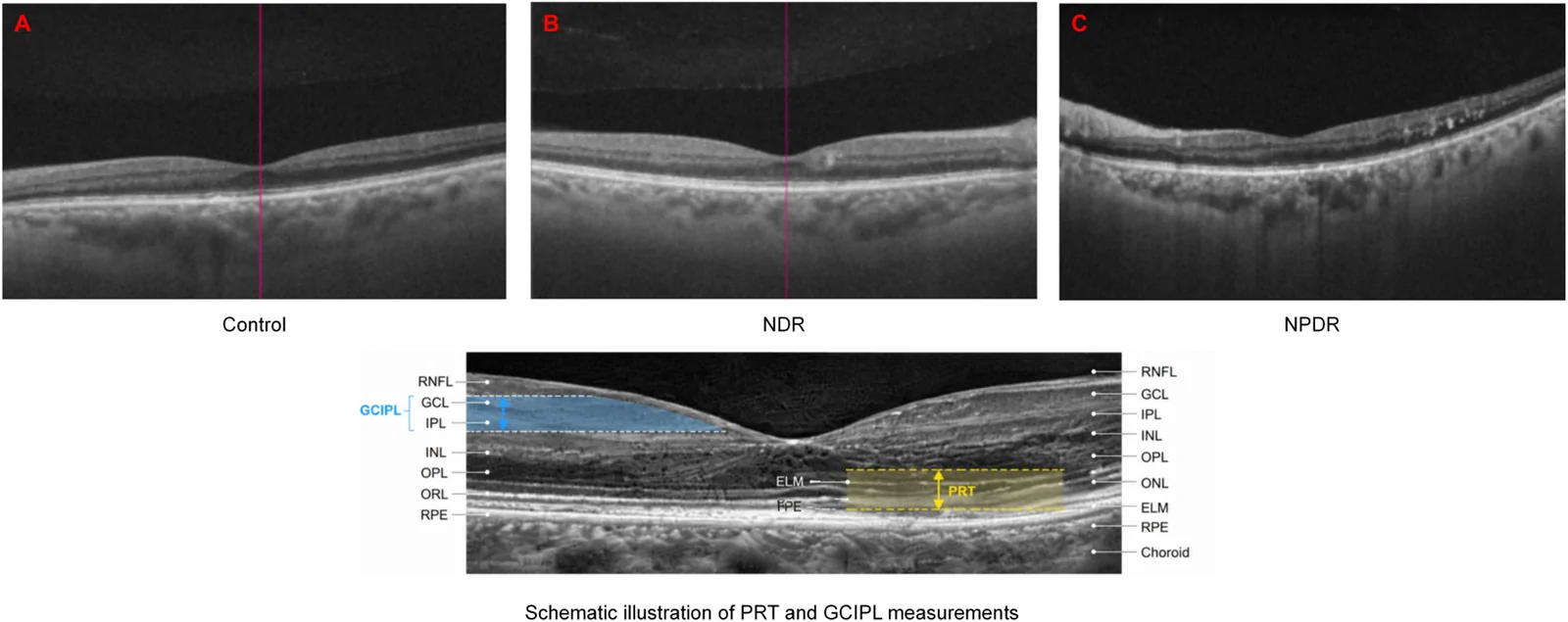
Representative OCT images and schematic illustration of retinal layer measurements. **(A)** Representative OCT B-scan from the Control group. **(B)** Representative OCT B-scan from the NDR group. **(C)** Representative OCT B-scan from the NPDR group. The schematic below illustrates the measurement locations of GCIPL and PRT.

Foveal avascular zone (FAZ) area and vessel density were automatically quantified from en-face OCTA images. Vessel density was calculated for the superficial capillary plexus (SCP) within a 6 × 6 mm region centered on the fovea.

#### 2.2.5 Statistical analysis

All statistical analyses were performed using R version 4.5.3. Continuous variables are presented as median and interquartile range (IQR), whereas categorical variables are presented as counts and percentages. Comparisons among the three groups were performed using the Kruskal–Wallis test, followed by pairwise Wilcoxon rank-sum tests with Bonferroni correction when appropriate. Categorical variables were compared using the *χ*^2^ test.

Pearson correlation was used as the primary correlation analysis, with Spearman correlation used as a sensitivity analysis. Benjamini–Hochberg false discovery rate (FDR) correction was applied for multiple correlation testing. In addition to the overall correlation analysis, exploratory subgroup correlation analyses were performed separately in the control, NDR, and NPDR groups.

Multivariable linear regression was performed among participants with T2DM to evaluate the association between photoreceptor layer thickness and middle-spatial-frequency contrast sensitivity. Model 1 was unadjusted; Model 2 was adjusted for age, sex, and axial length; Model 3 was additionally adjusted for T2DM duration and HbA1c; and Model 4, used as an extended sensitivity model, further included BCVA, hypertension, and hyperlipidemia.

ROC analysis was performed as an exploratory assessment of the ability of photoreceptor layer thickness to identify contrast sensitivity abnormalities among participants with T2DM in the NDR and NPDR groups. Abnormal contrast sensitivity was defined as a middle-spatial-frequency CS value below the empirical 5th percentile of the control-group distribution, corresponding to a cutoff value of 0.8235 in the present cohort. This threshold was internally derived from the control group and was not based on an externally validated reference value. The optimal PRT cutoff was determined using the Youden index, and sensitivity, specificity, positive predictive value (PPV), and negative predictive value (NPV) were calculated. A two-sided *P* value < 0.05 was considered statistically significant.

## 3 Results

### 3.1 Demographics

Demographic data for the participants are presented in Table 1. Ninety eyes from 90 individuals were included in this study. Of the total 90 participants, there were 30 individuals each in the control, NDR, and NPDR groups. Study groups presented no differences in age, gender, smoking, drinking, hypertension, hyperlipidemia, AST, ALT, and ophthalmic examination (including log MAR, BCVA, IOP, axis length, and lens status). Platelet counts were higher in the NPDR than NDR group, while neutrophil and lymphocyte counts were higher in NPDR than both other groups. Participants in the NPDR group had a significantly longer T2DM duration and higher HbA1c levels than those in the NDR group (Table 1 and Supplementary Table S4B).

**Table 1:** Demographic and clinical characteristics of study subjects.

Characteristic	Control (*N* = 30)^a^	NDR (*N* = 30)^a^	NPDR (*N* = 30)^a^	*p*-value^b^
Age (years)	59.0 (40.0–60.0)	43.0 (40.0–57.8)	56.5 (45.0–59.8)	0.051
Gender (Male)	19 (63.3%)	15 (50.0%)	15 (50.0%)	0.488
Smoking	14 (46.7%)	14 (46.7%)	17 (56.7%)	0.670
Drinking	19 (63.3%)	19 (63.3%)	22 (73.3%)	0.638
T2DM	0 (0.0%)	30 (100.0%)	30 (100.0%)	<0.001
T2DM duration (years)	NA	3.35 (1.42–6.02)	4.25 (2.10–8.53)	0.030
Hypertension	8 (26.7%)	14 (46.7%)	10 (33.3%)	0.257
Hyperlipidemia	8 (26.7%)	14 (46.7%)	10 (33.3%)	0.257
HbA1c (%)	NA	8.00 (6.08–9.28)	9.65 (6.08–11.18)	0.023
ALT (U/L)	16.55 (14.00–43.85)	30.15 (27.93–31.18)	16.95 (15.60–40.25)	0.890
AST (U/L)	26.55 (7.03–27.08)	19.20 (18.73–38.85)	35.40 (8.33–36.08)	0.079
Platelet count ( × 10⁹/L)	231.30 (222.25–266.20)	221.55 (211.80–249.70)	248.55 (228.08–280.63)	0.026
Neutrophil count ( × 10⁹/L)	3.86 (3.13–4.71)	4.65 (3.51–5.45)	6.48 (3.96–7.48)	<0.001
Lymphocyte count ( × 10⁹/L)	2.55 (1.95–3.24)	2.35 (1.70–3.06)	3.74 (2.48–4.50)	<0.001
BCVA	0.13 (0.13–0.13)	0.13 (0.13–0.19)	0.17 (0.10–0.18)	0.197
IOP	12.50 (12.3–17.8)	14.50 (11.9–17.0)	16.50 (15.8–16.9)	0.658
Axial length	23.79 (22.79–24.79)	23.45 (22.46–24.32)	23.15 (22.14–24.14)	0.171
Lens status				0.422
1	18/30 (60%)	20/30 (67%)	20/30 (67%)	
2	12/30 (40%)	8/30 (27%)	10/30 (33%)	
3	0/30 (0%)	2/30 (6.7%)	0/30 (0%)	

Lens status 1: lens; lens status 2: Intraocular Lens (IOL); lens status 3: without lens. T2DM, Type 2 diabetes mellitus; ALT, alanine aminotransferase; AST, aspartate aminotransferase; BCVA, best corrected visual acuity; IOP, intraocular pressure.

a Continuous variables are presented as median (IQR), and categorical variables as *n* (%).

b P values for continuous variables were calculated using the Kruskal–Wallis rank-sum test for three-group comparisons. T2DM duration and HbA1c were compared between the NDR and NPDR groups using the Wilcoxon rank-sum test. Categorical variables were compared using the *χ*^2^ test.

### 3.2 Measurement of CS

AULCSF and middle spatial frequency CS differed significantly among the three groups, whereas no significant differences were observed in low- or high-spatial-frequency CS (Table 2, Figures 3C,D). *Post-hoc* analyses showed that AULCSF was significantly lower across all three pairwise comparisons. Middle spatial frequency CS was significantly lower in both the NDR and NPDR groups than in the control group, whereas no significant difference was observed between the NDR and NPDR groups after Bonferroni correction (Supplementary Table S4A).

**Table 2:** CS analysis of study subjects. .

CS measure	Control (*N* = 30)^a^	NDR (*N* = 30)^a^	NPDR (*N* = 30)^a^	*p*-value^b^
AULCSF	1.13 (1.06–1.20)	1.05 (0.97–1.08)	0.91 (0.82–0.98)	<0.001
Low CS (logCS)	1.27 (1.22–1.40)	1.33 (1.24–1.39)	1.35 (1.23–1.42)	0.585
Middle CS (logCS)	1.33 (1.19–1.48)	1.13 (1.03–1.29)	0.99 (0.74–1.23)	<0.001
High CS (logCS)	0.81 (0.65–0.91)	0.74 (0.70–0.80)	0.71 (0.59–0.78)	0.130

Low-, middle-, and high-spatial-frequency CS correspond to 3, 6–12, and 18 cyc/deg, respectively.

a Median (IQR).

b Kruskal–Wallis rank sum test.

**Figure 3:**
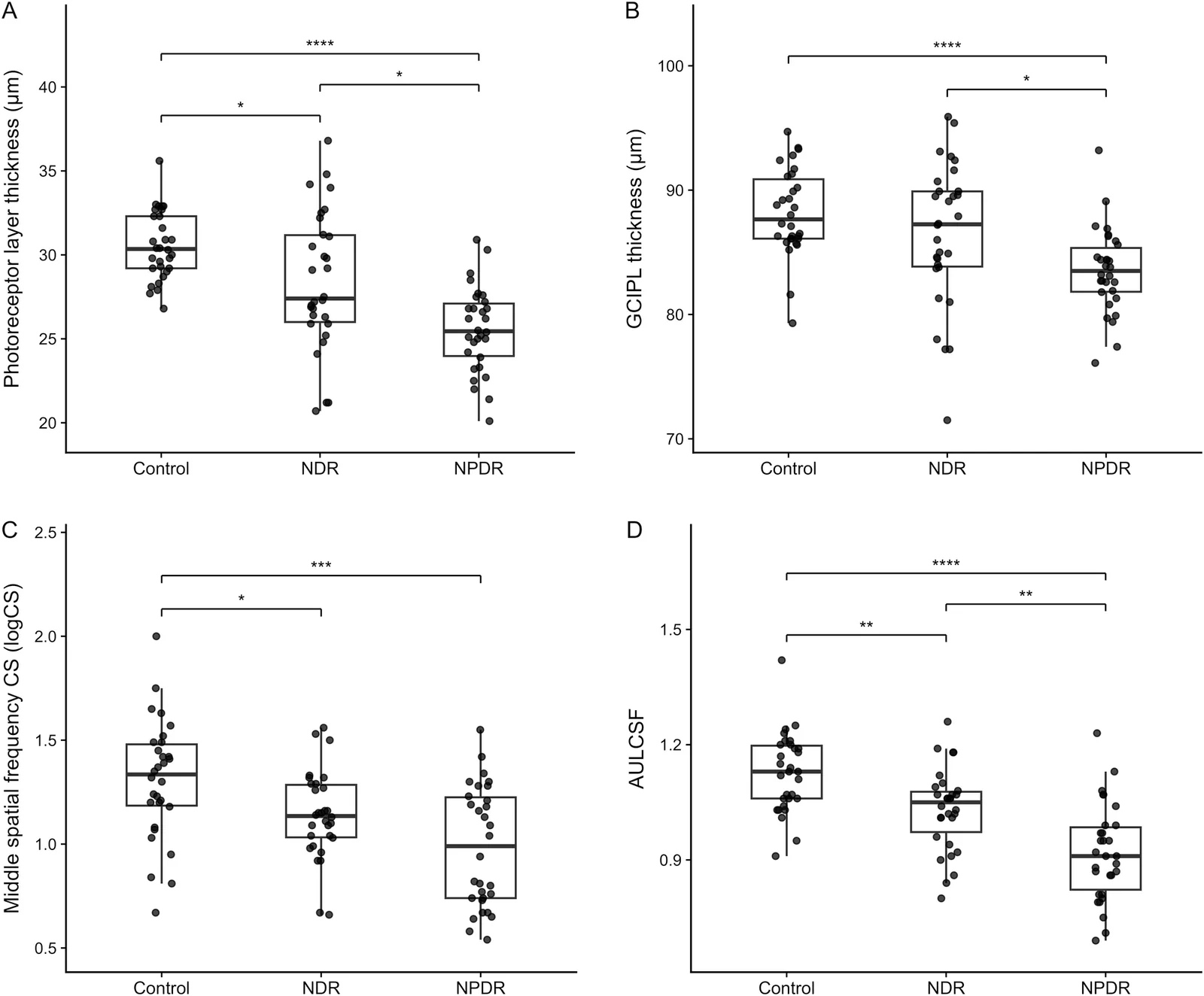
Comparison of retinal structural and functional parameters among the three groups. **(A)** Photoreceptor layer thickness. **(B)** GCIPL thickness. **(C)** Middle spatial frequency contrast sensitivity. **(D)** AULCSF. Data are presented as boxplots with individual data points. Statistical comparisons were performed using Bonferroni-adjusted Wilcoxon rank-sum tests. *P < 0.05, **P < 0.01, ***P < 0.001, ****P < 0.0001.

### 3.3 Retinal structure and blood flow

Representative OCT images from the control, NDR, and NPDR groups are shown in Figure 2. Photoreceptor layer thickness showed a progressive reduction across diabetic stages, with lower values observed in the NDR and NPDR groups compared with controls (Table 3, Figure 3A). Bonferroni-adjusted *post-hoc* analyses confirmed significant differences in PRT across all three pairwise comparisons (Supplementary Table S4A). In contrast, GCIPL thickness was significantly lower in the NPDR group than in both the control and NDR groups, whereas no significant difference was observed between the control and NDR groups (Table 3, Figure 3B). Exploratory analyses showed significant group differences in CD and CR. CD values were lower in the NPDR group than in controls, while CR values were lower in both the NDR and NPDR groups. Given the limited methodological documentation, the CD and CR findings should be interpreted cautiously. Overall, the PRT results suggest that photoreceptor layer thinning may occur before clinically detectable retinopathy develops (Table 3).

**Table 3:** Measurements of the retinal structure.

Retinal structural parameter	Control (*N* = 30)^a^	NDR (*N* = 30)^a^	NPDR (*N* = 30)^a^	*p*-value^b^
Total retinal thickness (µm)	230.2 (225.9–235.9)	228.1 (218.8–235.2)	227.4 (214.9–234.0)	0.257
Inner retinal thickness (µm)	60.0 (54.7–64.7)	56.8 (52.6–62.2)	55.5 (53.2–61.7)	0.282
Outer retinal thickness (µm)	161.5 (157.3–169.9)	165.6 (154.8–168.3)	162.1 (150.9–169.3)	0.650
Photoreceptor layer thickness (PRT, µm)	30.4 (29.2–32.3)	27.4 (26.0–31.2)	25.4 (24.0–27.1)	<0.001
GCIPL thickness (µm)	87.7 (86.1–90.9)	87.2 (83.8–89.9)	83.5 (81.8–85.3)	<0.001
CD (exploratory index)	18.3 (17.8–18.7)	18.1 (17.0–18.5)	17.4 (17.1–18.0)	0.002
CR (exploratory index)	92.5 (88.9–93.9)	87.0 (84.5–92.2)	86.6 (83.9–88.4)	<0.001

CD and CR were analyzed as exploratory indices. Their original measurement procedures and reporting scales could not be fully verified.

a Median (IQR).

b Kruskal–Wallis rank sum test.

The FAZ, VDs, and AFI of the SCP and DCP showed no significant differences among the three groups (Table 4).

**Table 4:** Measurements of the retinal blood flow.

Retinal blood flow parameter	Control (*N* = 30)^a^	NDR (*N* = 30)^a^	NPDR (*N* = 30)^a^	*p*-value^b^
FAZ area (mm^2^)	0.26 (0.21–0.36)	0.30 (0.21–0.40)	0.30 (0.25–0.38)	0.492
Vessel density of SCP (%)	54.50 (51.77–56.48)	51.61 (49.93–54.83)	53.90 (50.38–56.10)	0.059
Vessel density of DCP (%)	60.92 (57.27–62.30)	61.32 (58.06–63.31)	60.02 (57.89–62.61)	0.475
AFI of SCP (a.u.)	0.29 (0.23–0.33)	0.29 (0.24–0.32)	0.27 (0.24–0.31)	0.732
AFI of DCP (a.u.)	0.25 (0.21–0.28)	0.28 (0.24–0.32)	0.27 (0.23–0.30)	0.054

a Median (IQR).

b Kruskal–Wallis rank sum test.

### 3.4 Detection of inflammatory factors

Among the inflammatory indices, only PLR differed significantly among the three groups, whereas no significant overall differences were observed in SII or NLR (Table 5). *Post-hoc* analyses showed that PLR was significantly lower in the NPDR group than in both the control and NDR groups, while no significant difference was observed between the control and NDR groups after Bonferroni correction (Supplementary Table S4A).

**Table 5:** Calculation of blood inflammatory factors.

Inflammatory index	Control (*N* = 30)^a^	NDR (*N* = 30)^a^	NPDR (*N* = 30)^a^	*p*-value^b^
SII ( × 10⁹/L)	373.6 (273.0–450.4)	416.2 (342.3–573.2)	369.5 (310.5–624.2)	0.251
PLR (ratio)	89.3 (76.3–131.4)	100.6 (81.4–130.1)	66.4 (52.6–100.4)	0.003
NLR (ratio)	1.6 (1.2–2.0)	1.8 (1.4–2.5)	1.6 (1.2–2.6)	0.169

a Median (IQR).

b Kruskal–Wallis rank sum test.

### 3.5 Correlation between CS middle and associated clinical characteristics

Figure 4 showed that PRT (r = 0.385, *P* = 0.0024) and PLR (r = 0.273, *P* = 0.0346) were positively correlated with middle CS in Pearson correlation analyses. The association between PRT and middle CS remained significant after BH-FDR correction and was consistent in the Spearman sensitivity analysis (*ρ* = 0.364, *P* = 0.0043). In contrast, the weaker association between PLR and middle CS did not remain statistically significant after multiple-testing correction. These findings suggest that reduced photoreceptor layer thickness is associated with impaired middle spatial frequency contrast sensitivity in participants with T2DM.

**Figure 4:**
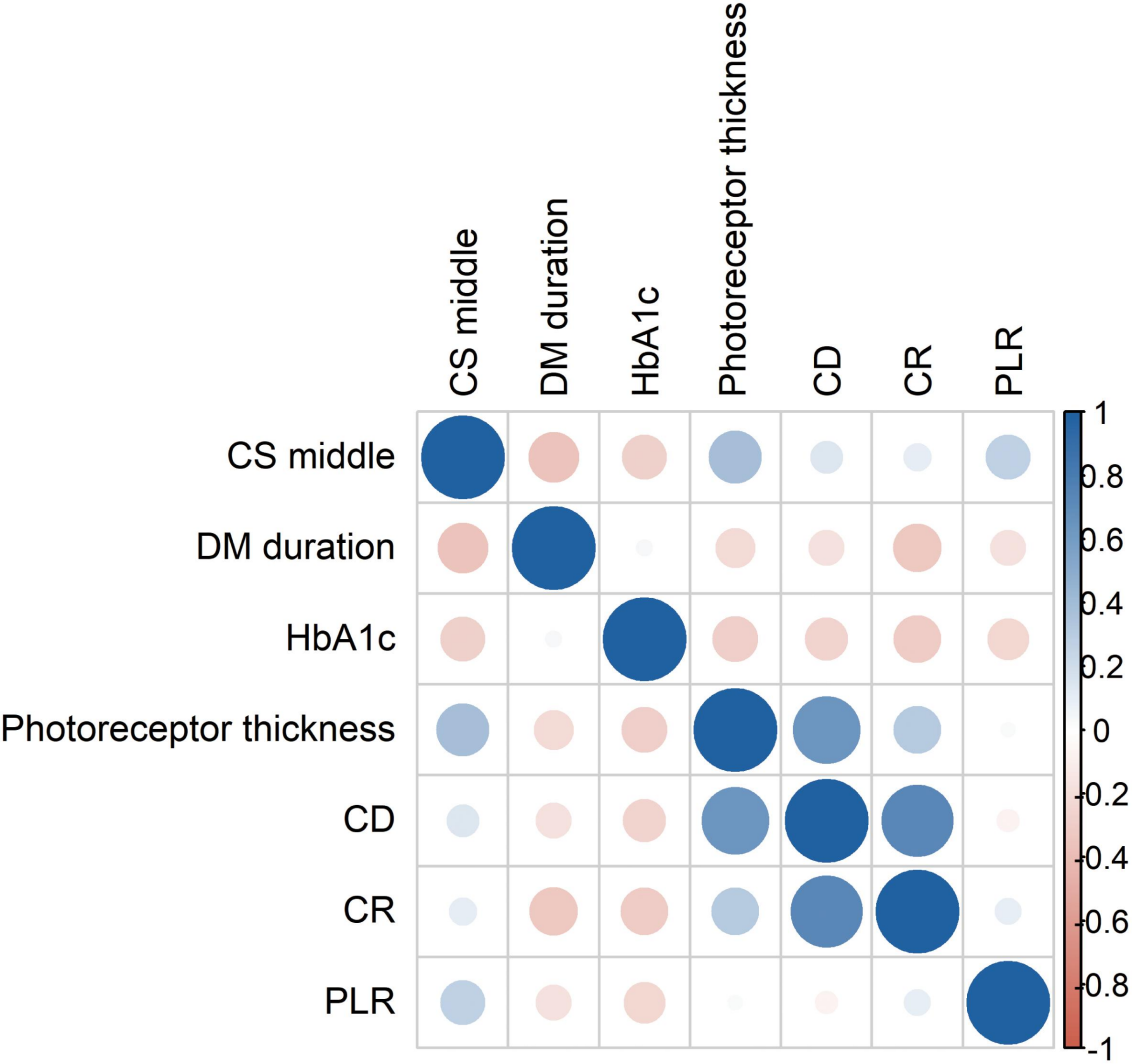
Correlation analysis between middle spatial frequency contrast sensitivity and retinal/clinical parameters in participants with T2DM. Correlation analyses were performed among the NDR and NPDR groups (*n* = 60). Blue indicates positive correlations, whereas red indicates negative correlations. Circle size and color intensity represent correlation strength. Pearson correlation coefficients **(r)** and exact *P* values for key associations are provided in the figure and/or described in the Results section.

To determine whether the observed association was influenced by differences among clinical groups, exploratory subgroup correlation analyses were further performed. The association between PRT and middle-spatial-frequency CS was not statistically significant in the control group (r = −0.207, *P* = 0.273) or the NDR group (r = 0.129, *P* = 0.498), whereas a significant positive association was observed in the NPDR group (r = 0.572, *P* = 0.001). Spearman analyses showed a similar pattern (Supplementary Table S2).

The association between PRT and middle CS remained statistically significant after adjustment for potential confounding factors. In Model 3, which included age, sex, axial length, T2DM duration, and HbA1c, PRT remained positively associated with middle CS (*β* = 0.0188, 95% CI: 0.0013–0.0362, *P* = 0.0354). The association remained significant in the extended sensitivity Model 4 after additional adjustment for BCVA, hypertension, and hyperlipidemia (*β* = 0.0205, 95% CI: 0.0023–0.0387, *P* = 0.0283; Supplementary Table S3). The direction and magnitude of the association were also consistent in the resampling-based sensitivity analysis.

### 3.6 The correlation between CS and PRT in patients with T2DM

Using the control-derived 5th-percentile cutoff for middle-spatial-frequency CS (<0.8235), 16 of the 60 participants with T2DM in the NDR and NPDR groups were classified as having abnormal CS, whereas 44 were classified as having normal CS. PRT differed significantly between participants with normal and abnormal CS (*P* < 0.001). Furthermore, Pearson correlation analysis revealed a positive association between PRT and middle-spatial-frequency CS (Figure 5).

**Figure 5:**
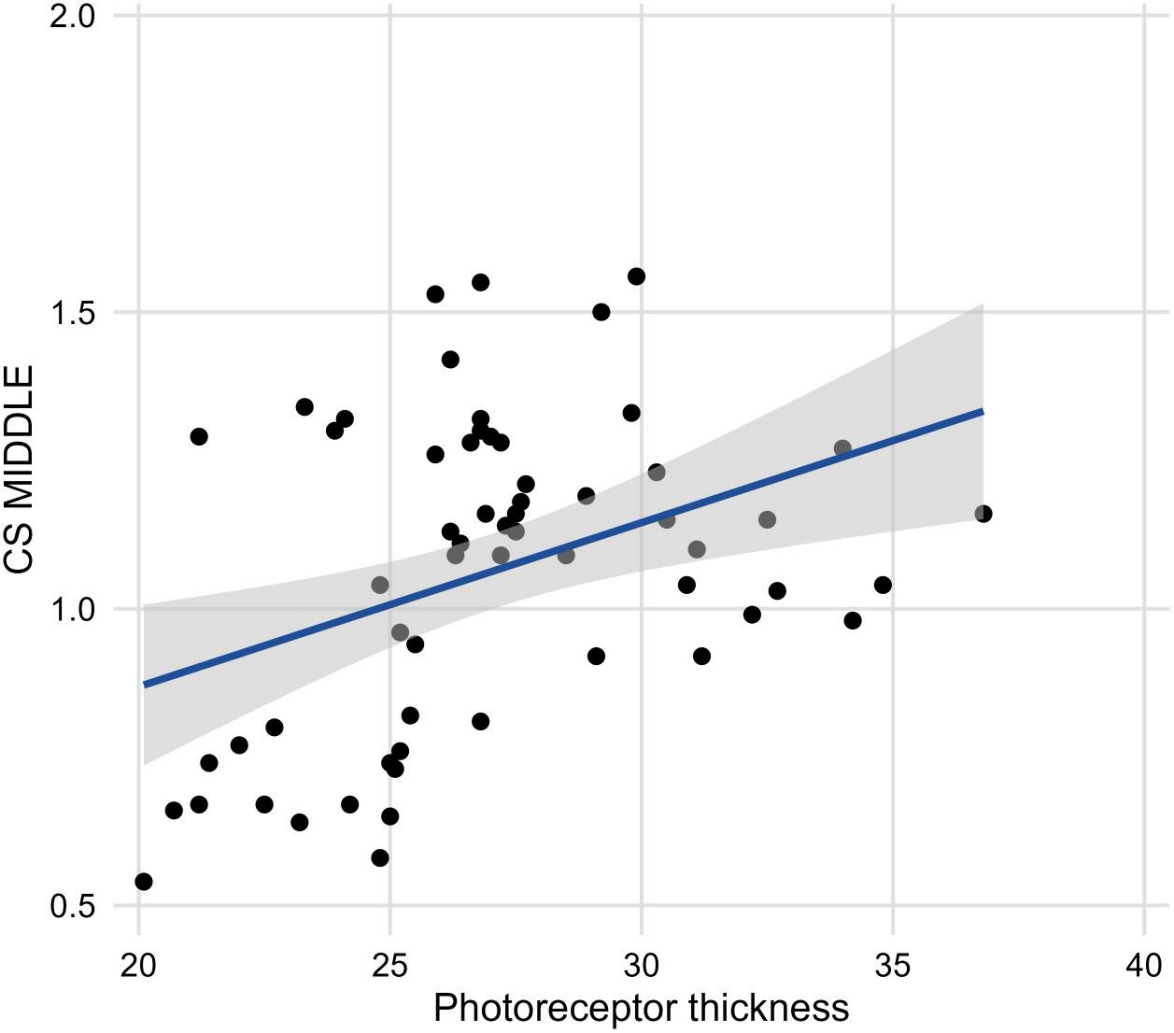
Association between photoreceptor layer thickness and middle spatial frequency contrast sensitivity in participants with T2DM. Each point represents one participant (*n* = 60). The solid line indicates the linear regression fit with 95% confidence interval. Pearson correlation analysis showed a positive association between PRT and middle spatial frequency CS (r = 0.385, *P* = 0.0024).

### 3.7 ROC curve analysis of photoreceptor thickness in patients with contrast sensitivity abnormalities

Exploratory ROC curve analysis was performed to evaluate whether photoreceptor layer thickness could discriminate participants with abnormal middle-spatial-frequency contrast sensitivity among those with T2DM. Using the control-derived threshold for contrast sensitivity abnormality, PRT showed potential discriminatory ability within the present cohort (AUC = 0.920, 95% CI: 0.852–0.989; Figure 6). The optimal PRT cutoff determined using the Youden index was 25.45 μm, with a sensitivity of 93.8% and a specificity of 86.4%. The corresponding PPV and NPV were 71.4% and 97.4%, respectively. However, this analysis reflects the ability of a structural retinal parameter to distinguish a functional test-defined outcome and does not indicate that PRT can diagnose diabetic retinopathy or predict future visual impairment.

**Figure 6:**
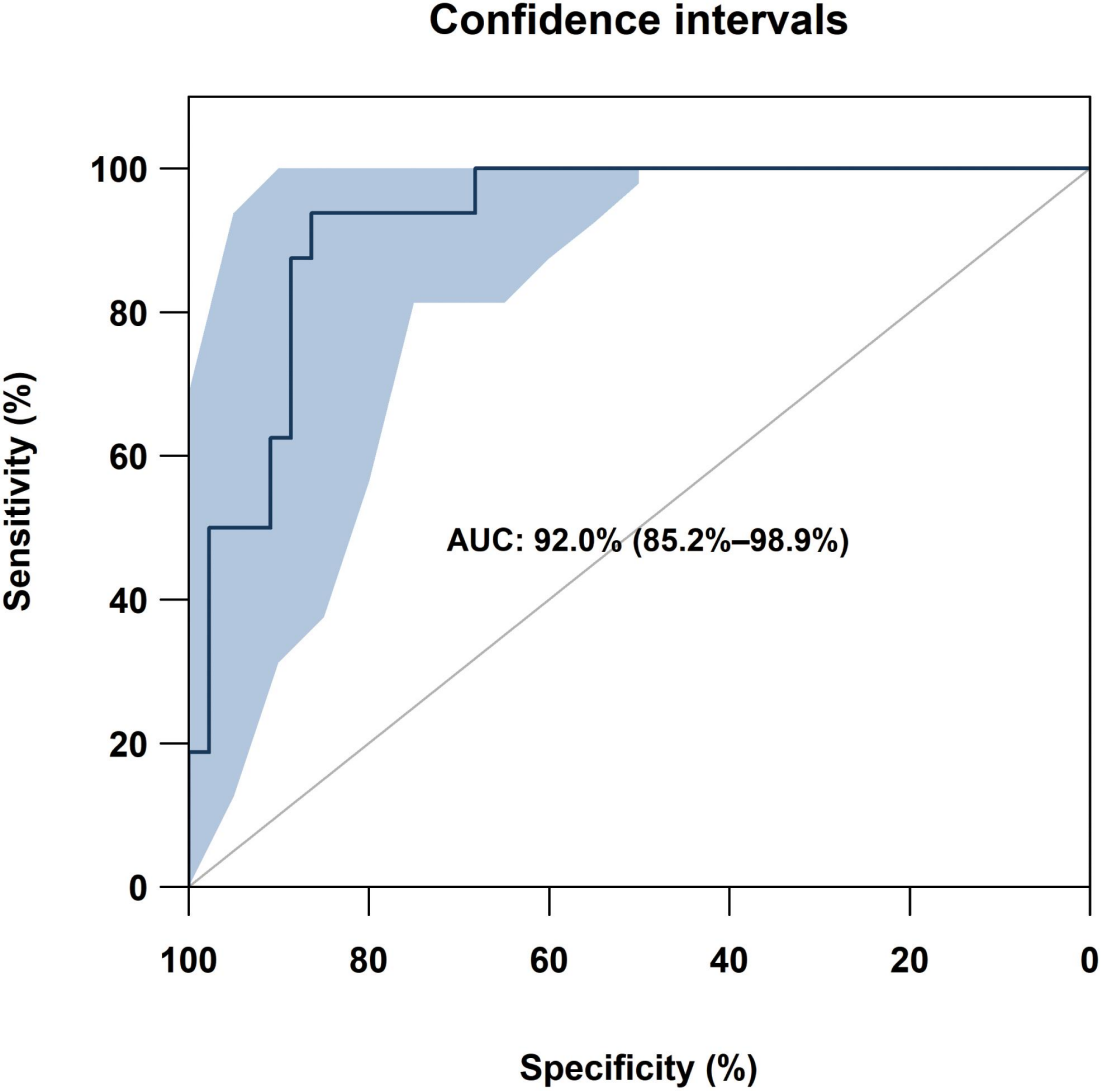
Exploratory ROC curve analysis of photoreceptor layer thickness for discriminating control-defined contrast sensitivity abnormalities in participants with T2DM.

## 4 Discussion

DR has been commonly considered a microvascular complication of T2DM. The pathophysiology of DR is complex; substantial evidence indicates that impaired communication between the nervous and vascular systems is a key factor in developing this condition (14). Despite the advancements in anti-VEGF medicines and photocoagulation for diminishing neovascularization and DME, numerous limits persist (4). Thus, researchers are now focusing on the early identification of DR.

CS means the black-and-white contrast of object surfaces necessary for the visual system to discern the spatial frequency of variously sized items, utilized to assess the resolution of the visual system concerning objects of differing dimensions (15). Clinically, form perception of the visual system is determined by visual acuity, which is usually measured at high contrast. However, CS may be independently compromised while visual acuity remains intact, complicating patients' ability to articulate their symptoms. Therefore, it may be particularly useful for detecting functional abnormalities in patients without apparent changes in visual acuity or fundus appearance. Several studies have reported alterations in CS among T2DM patients with or without DR (16, 17). Patients with non-insulin-dependent diabetes mellitus (NIDDM) and no retinopathy exhibit abnormal CS at only one spatial frequency (22.8 cyc/deg). In contrast, patients with NIDDM and background retinopathy show abnormal CS across all spatial frequencies (8). In the present study, we observed that CS was reduced in patients with T2DM before clinically apparent visual impairment occurred. Moreover, the level of AULCSF was also reduced in NDR and NPDR groups, indicating a decline in visual function within the central region of the macula. The precise mechanisms underlying the decrease in CS in individuals with T2DM and DR are not yet clear, and we will continue to explore this in the future.

DR is a multifaceted pathological process encompassing vascular and neuronal injuries (18). Photoreceptor cells, the specialized retinal neurons, transform light into neural signals, which are then transmitted to the brain for processing visual images. These cells are the most abundant in the retina, with an estimated 120 million in humans, and are also the most metabolically active cells in the body (19). The mechanisms underlying alterations in photoreceptor-related retinal structures in individuals with T2DM remain inadequately investigated; it is postulated that prolonged hyperglycemia, hypoxia, lipid dysregulation, oxidative stress, inflammatory mediators, photodamage, and compromised insulin signaling may contribute to these structural alterations (10). A previous study reported that the photoreceptor layer is thinner in people with diabetes than in healthy controls, while the thickness of GCIPL remained unchanged (20, 21). Similar results were also observed in our study. The PRT was reduced in the NDR and NPDR groups, whereas GCIPL levels remained unchanged in the NDR group and decreased in the NPDR group. Furthermore, early glycemic control has been reported to partially restore PRT (22), suggesting that changes in photoreceptor layer thickness may be detectable during the early stages of diabetes. Numerous studies have documented the associations between retinal function and microstructural changes in DR (23, 24). The present investigation revealed that reduced CS was associated with decreased photoreceptor layer thickness. Importantly, the association between PRT and middle-spatial-frequency CS persisted after adjustment for age, sex, axial length, T2DM duration, and HbA1c, and remained statistically significant in an extended sensitivity model additionally including BCVA, hypertension, and hyperlipidemia. These findings suggest that the relationship between photoreceptor structural impairment and contrast sensitivity reduction is unlikely to be fully explained by major clinical confounding factors. However, given the cross-sectional design, these findings should be interpreted as an association rather than evidence of causality. Because the OCT-derived PRT measurement did not include the ONL and did not directly quantify photoreceptor cell number, the observed reduction should be interpreted as thinning of the defined photoreceptor-related outer retinal compartment rather than evidence of photoreceptor cell loss. However, the contribution of individual photoreceptor subcomponents, including the outer segment, to the observed PRT reduction could not be determined because these components were not separately analyzed. The biological mechanisms linking photoreceptor structural alterations to contrast sensitivity impairment remain unclear. Based on previous studies, photoreceptor dysfunction may potentially influence retinal homeostasis through interactions with the neurovascular unit and blood-retinal barrier integrity; however, these mechanisms were not directly evaluated in the present study and should be considered as hypotheses requiring further validation.

A weaker positive association was also observed between PLR and middle spatial frequency contrast sensitivity in the unadjusted analysis. However, this association did not remain statistically significant after correction for multiple testing. Therefore, the relationship between systemic inflammatory status and retinal functional alterations should be considered exploratory and requires confirmation in larger studies. PLR, along with other systemic inflammatory indices like the Systemic Inflammation Index (SII) and Neutrophil-to-Lymphocyte Ratio (NLR), has been increasingly recognized as a predictor for diabetic microvascular complications, including diabetic retinopathy and nephropathy (25). Our results extend this association to the functional level, suggesting that systemic inflammatory status may be associated with early retinal functional alterations in patients with T2DM. Further studies are needed to clarify the underlying mechanisms. This potential mechanism warrants further investigation in longitudinal studies.

It was reported that the thickness of GCIPL was decreased as the DR stage progressed (26). We speculate that chronic hyperglycemia, even without noticeable clinical microvascular complications, may adversely impact retinal ganglion cells (RGCs), resulting in functional deterioration and eventual loss. Barber et al. demonstrated elevated apoptosis of retinal neural cells in both experimental diabetic rat models and human patients with diabetes (27). This process would likely lead to a decrease in the thickness of the GCIPL. Moreover, research indicates that although there is effective anatomical reduction of macular foveal thickness in patients with DME post-treatment, GCIPL thickness remains inferior to that of individuals with DR without DME and is associated with visual acuity. This further confirms the importance of GCIPL thickness in the neuro-ophthalmic manifestations of DR (28). A study comprised 895 individuals with T2DM, of whom 770 were categorized in the NDR group and 125 in the NPDR group, alongside 847 healthy control subjects. After a two-year follow-up, the loss of GCIPL was most rapid in the NPDR group, with a rate of −1.02 μm/year and −0.88 μm/year faster in the NDR group than the control group. Patients with T2DM experience a faster rate of GCIPL thinning (29). In our study, it was found that the thickness of GCIPL was reduced in the NPDR group, but the rate of decline was not detected. Hao et al. found that the thickness of GCIPL was positively correlated with the superficial retinal vessel density in the macular area (30). At the same time, there were no significant differences in FAZ, VDs, and AFI of SCP and DCP among the three groups in our study. In this cohort, no statistically significant differences were detected in the evaluated OCTA parameters, including FAZ, VD, and AFI. However, the absence of significant differences does not exclude subtle or early microvascular alterations, particularly considering the sample size and cross-sectional design. Therefore, whether neuroretinal structural and functional alterations precede microvascular changes requires further investigation in longitudinal studies.

Our study has several limitations. First, the sample size was relatively limited and may not fully represent the broader population of individuals with early-stage DR, particularly when considering specific subgroups. Although no significant differences in sex distribution were observed among the three groups, the relatively small sample size may have limited our ability to further evaluate potential sex-related differences in retinal structure and visual function. Larger cohorts are required to clarify these potential effects. Therefore, subgroup analyses according to T2DM duration, DR severity, or sex were not performed. Second, the ROC analysis was exploratory and was performed in the same relatively small, single-center cohort in which the CS abnormality threshold was defined. Therefore, the estimated AUC of 92.0% may be optimistic and should not be interpreted as established clinical diagnostic performance. External validation in larger, independent, multicenter cohorts is required. Thirdly, the cross-sectional design of this study restricts our ability to establish a definitive causal relationship between reduced CS and early retinal structural alterations. Fourth, there was a lack of detailed characterization regarding microaneurysm burden, precise macular involvement, and exact symptom duration, which limits deeper mechanistic interpretations. In addition, the lack of statistically significant OCTA differences should be interpreted cautiously, as subtle microvascular alterations may not be detectable in this relatively small cross-sectional cohort. PRT was manually measured by a single examiner without formal intraobserver or interobserver repeatability assessment. The examiner's masking status was not documented. These limitations may affect measurement reproducibility and introduce potential observer bias. The original measurement details of CD and CR could not be fully verified, including the analysis software, retinal region, automated processing procedure, and reporting scales. This limits their reproducibility and interpretation. Accordingly, these findings should be regarded as exploratory and require further validation using standardized imaging methods. Finally, the study lacked longitudinal follow-up and intervention data. In the future, we will continue to explore the effects of early diabetes control on the photoreceptor layer and CS.

In conclusion, our study demonstrates that functional visual deficits, specifically a reduction in middle spatial frequency contrast sensitivity, are present in T2DM patients even before the onset of clinically detectable diabetic retinopathy. Although previous studies have reported CS abnormalities in early T2DM, our findings further demonstrate an association between reduced contrast sensitivity and photoreceptor layer thinning using combined CS testing and OCT. These findings highlight the potential value of combining high-resolution OCT with quantitative CS testing for detecting early structural and functional retinal abnormalities in diabetes. Future longitudinal studies are warranted to determine whether these early structural and functional changes are associated with the subsequent development and progression of DR.

## Data Availability

The raw data supporting the conclusions of this article will be made available by the authors, without undue reservation.
